# Transforming cities for sustainability: A health perspective

**DOI:** 10.1016/j.envint.2020.106366

**Published:** 2021-02

**Authors:** Melanie Crane, Simon Lloyd, Andy Haines, Ding Ding, Emma Hutchinson, Kristine Belesova, Michael Davies, David Osrin, Nici Zimmermann, Anthony Capon, Paul Wilkinson, Catalina Turcu

**Affiliations:** a Sydney School of Public Health, The University of Sydney, Australia; b Centre for Climate Change and Planetary Health, London School of Hygiene and Tropical Medicine, United Kingdom; c Bartlett School of Environment, Energy & Resources, University College London, United Kingdom; d Institute for Global Health, University College London, United Kingdom; e Bartlett School of Planning, University College London, United Kingdom

**Keywords:** Cities, Sustainability, Liveability, Environmental Health, Urban population, Urban policy, Governance, Urban transformation, Urban planning, Systems science

## Abstract

Transformational change is urgently needed to address planetary health challenges in cities. Through an interdisciplinary overview of the literature, we consider how to frame and unpack city-level transformation towards synergistic benefits for urban health and environmental sustainability. By describing the characteristics of a ‘healthy sustainable city’ and by bringing together the ideas underlying frameworks for health and sustainability, we develop a conceptual understanding of how cities may progress towards achieving significant improvements in health and the environment. We investigate how urban change works, and build a theoretical understanding of how urban change may be directed to integrate health and sustainability. We conclude that urban transformation needs to be a multi-scalar process across city sectors to meet the scale, speed and form of change required. We propose that this can best be achieved in practice through a composition of mechanisms, including strengthening city governance, enabling technological and social innovations, applying sustainable urban planning and infrastructure development, and impelling social behaviour change; supported by systems-driven policy and practice-focused scientific evidence.

## Highlights

• To safeguard human and planetary health, urban transformation is needed.
• Urban transformation must be at a pace and scale not previously undertaken.
• It requires ambitious, integrative city-level actions for health and the environment.
• Changes in political, social and economic systems will be necessary to accelerate city actions.
• System science, urban structures and processes are needed to drive innovative action.

## 1 Introduction

Transformative change in cities is needed to address the current and future challenges to health and sustainability posed by the far-reaching environmental trends occurring in the Anthropocene epoch (Steffen et al., 2018). Environmental sustainability and human health are interwoven and there will be mutual benefits if cities can address both needs through integrated approaches (Haines et al., 2009). This paper sets out to determine how change in urban settings could be brought about to achieve health and environmental goals synergistically, taking into account the potential trade-offs of focusing exclusively on either health or environmental issues. Compartmentalised approaches to research and policy, in which sectors and disciplines often work in silos, have been characteristic of modern industrialized societies. The current situation requires a planetary health approach, integrating actions to promote and protect the health of populations and the state of natural systems on which health ultimately depends (Whitmee et al., 2015). This paper therefore sets out to determine the actions needed to achieve health and sustainability and how these can be integrated and implemented at the speed and scale required. The insights have arisen from an international city research – policy partnership, Complex Urban Systems for Sustainability and Health (CUSSH), that aims to help shape policy decisions and their implementation for health and sustainability in urban settings. We begin by considering the importance of cities, the drivers of change within them and the opportunities they offer. We examine the available frameworks for bringing together sustainability and health and critique their limitations, including their lack of a transdisciplinary perspective. We propose what transformational change might look like and outline potential approaches for accelerating action.

## 2 What is known about planetary health

### 2.1 Cities are central

Urbanisation is occurring at a tremendous pace and scale. Cities are home to about 55% of the world’s population, a figure projected to increase to 68% by 2050 (United Nations, 2018). Most of this growth is occurring in emerging cities in Asia and Africa (United Nations, 2018), where the opportunities are therefore greatest to influence actions for health and sustainability at an early stage of urban growth and development. However, action is needed also established cities, to tackle environmental concerns, through reducing carbon emissions, improving climate resilience and protecting natural ecosystems (Steffen et al., 2018), which will often also provide health gains (Whitmee et al., 2015, Watts et al., 2015). Cities offer opportunities for synergies between health and environmental actions because of the concentration of collective economic, social and technological capacities for innovation. However, urbanisation is also crucial driver of environmental impact, with detrimental consequences for human and natural systems at a planetary scale (Whitmee et al., 2015). For instance, urban development influences the risks of communicable and non-communicable diseases, malnutrition, injuries, and vulnerability to global environmental changes (Whitmee et al., 2015, Prüss-Ustün et al., 2016, Frumkin and Haines, 2019). Susceptibility to these risks is moderated by social and demographic factors, with considerable inequities across cities and countries and between deprived and wealthy neighbourhoods within cities (MacInnes et al., 2015). A key objective underlying the achievement of sustainable cities is to shift the balance away from these harms (and inequities) towards the achievement of multiple benefits.

Many urban health and environmental challenges to be addressed are a consequence of how we organize and live in cities (Dodman, 2009). These factors span multiple sectors, including energy, housing, transportation, planning, agriculture, water and waste (Ramaswami et al., 2016). Energy use in cities relies heavily on fossil fuels, accounting for 70% carbon emissions worldwide, meaning energy transitions to renewables could play a key role in emissions reduction (Edenhofer, 2015). While cities are responsible for the largest share of carbon emissions, substantial differences exist between cities and within countries, because of lifestyle choices (shaped by within-city contexts) and land use decisions, relating to urban infrastructure (Hoornweg et al., 2011). For example, emissions from the transport sector are disproportionately high in cities with low urban density and substantial urban sprawl; here, densification - with cities growing within smaller boundaries instead of outwards - is central to minimising impact (Edenhofer, 2015). High levels of food and water consumption in cities draw on resources from urban hinterlands, which, along with urban expansion, place pressure on local food and water security (Vanham et al., 2017, Opitz et al., 2016). Increased consumption has brought increased waste production. The World Bank estimates that, given current trends, 3.4 billion tonnes of waste will be generated annually by 2050: a 70% increase on current volumes (Kaza et al., 2018). This amount of waste could largely be eliminated in cities through a circular economy, but requires major changes in structures and processes across many sectors (Rizos et al., 2017).

### 2.2 Urban solutions are co-dependent and inter-connected

**Fig. 1:**
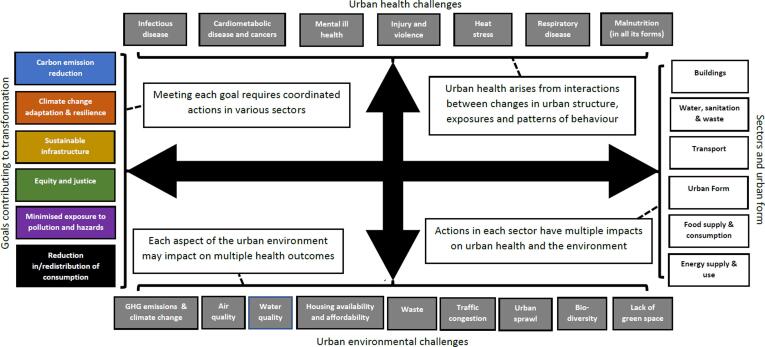
A framework illustrating the urban conditions and cross-sectoral actions needed to achieve urban transformations for sustainability*. A list of some of the key urban health and urban environmental challenges are included along the top and bottom. The left side of the figure shows some selected co-beneficial goals for environmental sustainability and public health. Initiatives taken to achieve a particular goal require actions to be taken in multiple sectors and on urban form (right side), and that each of these actions will yield multiple health and environmental benefits if they are well designed.

The salient issues for achieving urban sustainability are co-dependence and interconnectivity: actions within a given sector are likely to impact on other sectors, and, achievement of any particular sustainability goal is likely to require actions across multiple sectors (Fig. 1) (Dora et al., 2015, Gao et al., 2018). For example, within-sector changes that shift a city’s energy supply from fossil fuel to low-carbon renewables may provide environmental benefits via reduced carbon emissions and improved air quality while simultaneously bringing health benefits, such as reduced premature cardio-respiratory deaths (as well as potentially fewer climate change-related health impacts in the future) (Buonocore et al., 2016). An integrated approach to address health and environmental challenges is necessary, not only because of interconnectivity but also because urban policies involve trade-offs, with potential unintended and unanticipated adverse consequences. For example, some urban tree species emit volatile organic compounds which can trigger health conditions, and growth in active travel can be accompanied by an increase in road injuries. Yet, despite calls for a combined approach to inform city-level change towards improvement in public health and environmental sustainability, achievements to-date have been limited (Heikkinen et al., 2019, Watts et al., 2018).

### 2.3 Action lies with cities

Cities (or rather, their political leaders, organizations, and citizens) are increasingly identified as agents for urban change. Cities are a major entry point for inter-sectoral public health programmes, sustainability and climate change action (World Health Organization, 2015, United Nations, 2017), potentially bypassing barriers to national level policy and decision-making (Hoornweg and Pope, 2017). Many cities are already politically committed to sustainable development and participate in urban networks for global action. City networks reach beyond geographic boundaries to influence identification, development and implementation of strategies for improving sustainability and health. However, *transformative* changes at the scale, speed, and form required to safeguard both human and planetary health have not, as yet, been accomplished (Heikkinen et al., 2019, Hölscher et al., 2019). Several initiatives have focused on specific objectives, for example, the *100 Resilient Cities* initiative has focused on adaptation and resilience to a range of shocks, the *Healthy Cities Movement* has focused on achieving equitable health improvements and the *C40 Cities* on climate change mitigation. Yet, there are few examples of integrated actions that aim to address equity, health, adaptation and mitigation of climate change and other threats to planetary health.

Fig. 1 outlines six key goals or conditions necessary to achieve planetary health: (1) Reduction in greenhouse gas emissions and drivers of other global environmental changes; (2) Adaptation and resilience to climate and other environmental change; (3) Sustainable urban infrastructure development; (4) Pursuit of equity and justice; (5) Environmental health protection by minimisation of pollution and hazards; and (6) Reduction in consumption with movement towards a circular economy. Together these offer a comprehensive approach to addressing urban health and environmental changes across sectors. The figure is intended to be illustrative and does not provide an exhaustive list of all challenges, goals, actions, outcomes or cross-linkages. It was derived from an overview of urban health and environmental challenges and primary goals collated from existing literature and a workshop with experts from across multiple disciplines, policy and industry representatives (Supplementary file – Table S.1).

## 3 Integrating health and sustainability

### 3.1 Current framing of health and sustainability

We overview how conditions for planetary health have been presented in the literature and practice. According to the World Health Organization (WHO), a healthy city is one that is “continually creating and improving physical and social environments… which enable people to mutually support each other in performing all the functions of life and developing to their maximum potential” (World Health Organization, 1998). These ideas have moved beyond fundamental sanitary aspects of health (water quality, waste containment and hazard reduction), which still remain challenges in many cities, to factors within the city that impact wider wellbeing (Pickett et al., 2013). The WHO Healthy Cities narrative embraces the importance of social-spatial and physical dimensions of cities for health (World Health Organization, 2015); with health and wellbeing broadly defined on physical, psychological and social levels (World Health Organization, 1948). Some definitions of healthy cities focus on communities within cities. Dannenberg et al. (2003) defined a healthy community as one that protects and improves the quality of life of its citizens, promotes healthy behaviours and minimises hazards for its residents (Dannenberg et al., 2003). More recent conceptualisations of cities focus on ‘liveability’, defining health and wellbeing through a social determinants framework to include aspects such as crime and safety, social cohesion and local democracy, education, employment and health services, housing affordability, public places for culture and leisure and nature (Badland et al., 2014). However, the healthy city concept does not fully encompass sustainability: goals focus on health of the current population, without an eye on future generations.

The term ‘sustainable city’ is used interchangeably with concepts such as the ‘green city’, ‘eco-city’ and ‘low-carbon city’. These concepts share an overall objective of reconciling environmental, social and economic goals, with some differences (De Jong et al., 2015). The ‘sustainable city’ is closely linked to the definition of sustainability put forward by the Brundtland Commission: “development that meets the needs of the present without compromising the ability of future generations to meet their own needs” (WCED, 1987), as well as to the “triple bottom line” (the three pillars: economic, social and environment of sustainable development) (Pickett et al., 2013). In most cases, interpretations of sustainable cities favour the importance of the environment, though some emphasise the socio-economic dimension (De Jong et al., 2015, Tanguay et al., 2010). Sustainable policies such as decarbonisation of the energy system, ecological conservation, are ultimately about improving living conditions to support human needs for opportunity, security, autonomy, wellbeing and health, without undermining the natural systems on which human civilisation ultimately depends. Sustainable policies aim to transform living, either through mitigation or adaptation actions, to optimise conditions in a way that can be maintained (McMichael et al., 2003).

Marrying the concepts of healthy and sustainable cities will enhance the ecological focus of health which has been largely missing from health promotion to date (Butler and Friel, 2006, Hancock, 2011), and the health focus which has been missing or underplayed in the urban sustainable development discourse (Haines et al., 2009, Dora et al., 2015). The United Nations (UN) Sustainable Development Goals (SDG 11) target on sustainable cities addresses social and environmental health needs such as adequate housing, poor air quality and hazardous waste disposal, while addressing health as a separate goal (SDG 3) (United Nations General Assembly, 2015). A number of the other goals address sectoral policies with major effects on health (Dora et al., 2015), including those focusing on the reduction of poverty, improvement of nutrition, provision of safe water and sanitation, and access to clean renewable energy (SDGs 1, 2, 6, 7). More broadly, the UN New Urban Agenda (NUA) encompasses what cities need to achieve for sustainability, although it fails to connect with health outcomes (United Nations, 2017).

Initiatives that address health promotion, the 1986 Ottawa Charter for Health Promotion and the later Sundsvall Statement emphasise the importance of a supportive environment for health (Poland et al., 2011). This encompasses physical, social, economic and political spaces where people live, work and play, which for most people entail the city. However, ‘city’ boundaries do not end at the border of the built environment: the environmental footprints of cities vastly exceed their physical boundaries and, vice versa, human health in cities is dependent on the sustainability of the planet and our ability to remain within the planetary boundaries that define a “safe operating space for humanity” (Steffen et al., 2015). This is important because, for example, as much as two-thirds of carbon emissions associated with consumption in cities may come from outside the city and are not recorded in sector-based impact inventories (C40 Cities Climate Leadership Group, 2018). Boyden illustrates the connection between human health, natural systems and the biophysical and cultural arrangements of human society as tri-interdependent (Boyden, 2011, Hoornweg et al., 2011). By implication, actions to improve the health or sustainability of cities are interconnected with the natural world, this necessitates development without further harm to the biosphere. Therefore, health promotion needs to work in parallel with sustainability goals directed at managing natural resources for current and future generations.

### 3.2 Review of current frameworks for health and sustainability

A number of conceptual frameworks have brought together common drivers for health and sustainability within the city context. Key aspects of these frameworks include: the importance of built and natural environments (Barton et al., 2015); the need to consider both equity and efficiency (Elmqvist et al., 2019); the various scales at which processes occur (Northridge et al., 2003); the role of urban policy and planning (Giles-Corti et al., 2019); complexity arising from interconnectivity (e.g. feedbacks, non-linearities) (Rydin et al., 2012); and the wider context which cities both influence and are influenced by (Ramaswami et al., 2016, Bai et al., 2016). This list reflects the fact that cities are complex socio-ecological systems with interdependencies between different parts and sub-systems and therefore need to be conceptualised as such. Existing conceptual models are firmly rooted in either sustainability or health studies, with few cutting across and discussing the intricacies and politics of interdependencies. Existing definitions and models do not bring health, sustainability and cities together in an explicit way, but rather signpost a number of aspects of mutual interest, including variation across spatial scales and contexts, the importance of power and democracy, social equity and social justice.

**Fig. 2:**
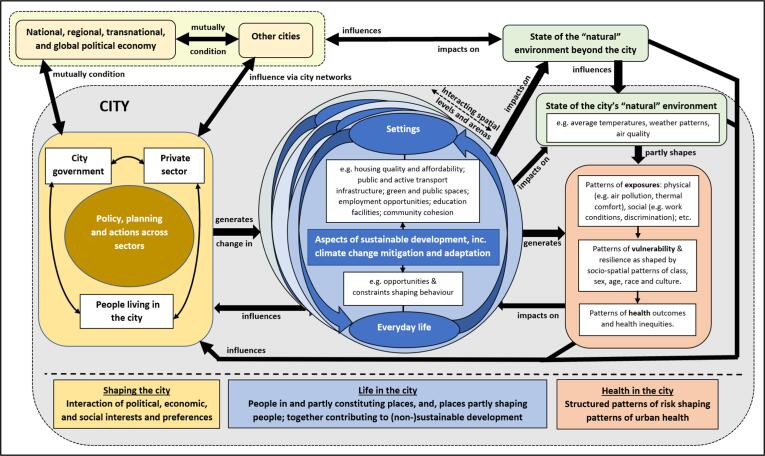
**A conceptual diagram of how cities impact on population health, sustainable development, and the “natural” environment by shaping life in the city.** The shaded grey area represents the city, with the dashed border indicating that cities have strong relations with places and processes beyond the city. The middle section represents ‘life in the city’, which involves mutually conditioning interactions between individual people and settings, which occurs at different spatial scales and in different arenas. The latter processes contribute materially and culturally to sustainable (or non-sustainable) development, including mitigation of and adaptation to climate change, and, they impact directly on the environment of the city including the “natural” environment, both within (e.g. local air quality, noise, ambient temperatures) and beyond the city (e.g. climate change). Living conditions, the state of the environment and its long term sustainability, and patterns of health and health inequities influence interests regarding city priorities for change, with the latter being the outcome of conflict, negotiation, and cooperation between the city government, the private sector, and people living in the city (as individuals or collectives). City priorities are also conditioned by (and condition) extra-city influences, via city-to-city networks and national, regional, transnational (e.g. via trade agreements), and global processes.

Lacking a clear definition of what a ‘healthy and sustainable city’ is, we propose a succinct definition as ‘a city that enables all people, communities and natural systems to thrive now and into the future’*.* This definition acknowledges the various scales and complexities of the city’s physical, social-economic and ecological dimensions within the wider biophysical region and planetary context. Fig. 2 attempts to conceptualise these ideas in a generic city, showing that “life in the city” is shaped by both people and place, and how this in turn impacts on local and global environments and generates patterns of population health, and how all these conditions then influence city priorities for health and sustainability. These priorities are ultimately determined and enacted following negotiations between the local authority, private enterprise, and citizens, but are also influenced by bi-directional relations that extend beyond the city, including via city-to-city networks, national conditions, transnational relations (such as treaty obligations), and global processes (e.g. negotiated carbon emission reduction commitments).

## 4 Achieving healthy sustainable cities requires transformational change

### 4.1 What transformational change for health and sustainability looks like

What a ‘healthy and sustainable city’ comprises and how it is achieved is the critical issue for city transformation. Given the potential benefits from aligning the health and environmental sustainability agendas in cities, we consider how cities might move towards this potential alignment; specifically, the drivers, actors, mechanisms and processes that underpin this change. Change towards sustainability in cities often falls largely into two broad categories: actions that bring about *transitional* change and actions that lead to *transformational* change (McCormick et al., 2013). Definitions of, and distinctions between, these concepts vary widely in the literature, reflecting differences in underlying theories of how change occurs (Feola, 2015, Meerow et al., 2016) and the specific concerns of different research fields (Hölscher et al., 2018). Of key importance is that actions are taken which catalyse change at the *speed* and *magnitude or scale* required to address the pressing environmental imperatives for planetary health, especially climate change (Watts et al., 2018), as well as local health and health equality needs amenable to environmental intervention.

Urban transformation necessitates fundamental changes to urban systems (Elmqvist et al., 2019, McCormick et al., 2013, Walker et al., 2004, Pelling et al., 2015, Ernst et al., 2016), targeted at the system structures (i.e. social and political arrangements, physical infrastructure and technology) and functions (Koch et al., 2017, Koch et al., 2017). Attempts to advance urban sustainability to date have not been transformative in pace and scale (Heikkinen et al., 2019). For example, schemes for the development of neighbourhood walking and cycling to promote health benefits of increased physical activity while supporting environmental benefits of low-carbon transportation have mostly been on a small scale, achieving only modest impact Local changes to existing systems of this kind may be easy to implement (and are thus more readily adopted by city planners), but they fall far short of the fundamental system-widechanges needed for effective climate action (Steffen et al., 2018, Messerli et al., 2019).

Deliberate, planned, purposive and very ambitious actions are needed across all sectors, spatial scales, and social and policy arrangements (McCormick et al., 2013, Feola, 2015). For example, (Loo and Tsoi, 2018) highlight five key changes needed to achieve transformation in the transport sector (Loo and Tsoi, 2018), spanning, infrastructure changes, city development and land-use planning policies, economic transition, vehicle/fuel technology and lifestyle changes. The required whole-system change can only be achieved through long-term and dedicated commitment and now must be brought about with increasing urgency. Copenhagen’s ambition to become carbon neutral by 2050 is only possible because of decades of planning with sustainability at the top of the agenda. Most other cities are still far behind and will have to achieve a similar ambition in little more than two decades if collective global action is to meet the goals of the Paris Climate Agreement. How cities will achieve these changes will differ from city to city depending on their current context and urgent priorities but will need wide-ranging and multiple, concerted action by both city and national authorities.

### 4.2 What theories are available to inform transformative change for health and sustainability?

In urban studies, the predominant contribution to transformation comes from two schools of thought: resilience theory and socio-technical studies. Resilience theory suggests that change is a response to disruption in a system, with resilience as the adaptive or transformative capacity to adjust to it (Holling, 1973). Social-ecological resilience, introduces the human dimension into systems thinking, focusing on the dynamics of change in complex social-ecological systems necessary for operating sustainably (Folke et al., 2010). A key theme of socio-ecological resilience is creating adaptive capacity for change, although this can be incremental as much as transformative. In the urban system this includes socio-economic factors, urban infrastructure and form, material and energy flows and governance, such as through planning and policy (Meerow et al., 2016). At the city-level this means working through a city’s political, social and business structures to make changes to physical infrastructures, such as conservation policies and watershed protection (Grandin et al., 2018), and may require reconfiguring decisional power to initiate and enable change (Olsson et al., 2014). However, resilience theory alone does not address the need to reduce the environmental footprint of cities. Nor does current urban resilience research address urban health, except inasmuch as social resilience provides the potential to expand the focus to encompass health. This is an oversight because well designed adaptation and resilience strategies have the potential to improve and protect health.

Socio-Technical Studies (STS), particularly the area of urban sustainability transitions, conceptualise urban change through four broad approaches: Transition Management, Technological Innovation Systems, Strategic Niche Management and the Multi-level Perspective (Markard et al., 2012). Geels (2005) describes urban change as shifts from one socio-technical system to another, involving technological innovation alongside other changes such as changes in social norms, market rules or institutional structures, and occurring over a long period (Geels, 2005). Resistance or inertia to change is attributed to rigid organisational, market and political structures and processes and embedded culture and social forces that act to “lock-in” existing systems (Pickett et al., 2013, Markard et al., 2012, Childers et al., 2014). Driving urban change requires coordination of bottom-up and top-down action, via integration of horizontal and vertical lines of power. Three interrelated levels at which socio-technical changes occur are (i) the micro or niche level (localised innovations); (ii) the meso or ‘regime’ level, representing the institutional structure of the system; and (iii) the wider or macro socio-technical landscape, or exogenous environments. The city governance ’regime’ and its role in the functioning of society is gaining attention as the central space for sustainability interventions; this includes city governments and other civil or business groups operating at the city-scale, (Köhler et al., 2019). Our focus on the city-scale recognises that micro- level innovations or interventions are in themselves embedded within the wider social, economic and political system of the city, and macro landscape and structural powers such as global pressures for change. A number of gaps in STS are recognised including the problem of how to scale-up niche interventions to the city level (Grandin et al., 2018). Moreover, STS see change happening progressively by putting pressure from the ‘bottom’ (niche), ‘making’ it into the regime (and changing predominant institutional structures) and, finally, being integrated at the macro-level of landscape. This it is not always the case for health-related change, where, for instance, public health change is required from the ‘top’ (regime or landscape level) to be implemented at the local level (niche).

These urban theories infer that social and political feasibility determines much of the potential for urban form or process changes. Urban governance refers to multiple actors that intervene directly or indirectly in the operation of the city, including formal government structures, industry, civil society and community groups. Governance, even as informal structures, is an important mechanism for coordinating systemic changes in cities. It can address the balance of selecting pressures (exerting change) and condition the adaptive capacity of the city system (Smith et al., 2005). Within the STS literature, Transition Management is a growing area focused on facilitating change through governance. A central objective is on bringing together the various city-level actors including policymakers, social and industry groups and research institutes to implement changes for sustainability or health (Köhler et al., 2019). The key characteristic of these actors is their capacity to make or influence city-level strategies (Farla et al., 2012). In examining the politics of rapid urban transformation to address climate change, Grandin and colleagues note that collaborative arrangements provide opportunities to align interests and mobilize actors, by moving common goals up respective agendas (Grandin et al., 2018). Processes by which urban governance may influence urban environmental action include political leadership collaboration between cities, coordination and institutional integration, public–private partnerships and political culture, community and science (Broto, 2017, Frantzeskaki et al., 2016).

In addition to urban theories, the wider literature on system science provides some universal principles of how systems operate and change, which have implications for how we think about transforming cities. In systems thinking, influencing the processes that act to support or oppose change requires change to the cause-and-effect feedback pathways (causal loops) of how a system operates. A simple example is that increasing the extent to which the urban design promotes active and public transport will decrease individual dependence on private motor vehicles (Proust et al., 2012). However, for a change to be transformational, it needs to go beyond simply changing the flow of processes made by constituents of the city, to address root causes (i.e. worldview, knowledge, beliefs and priorities and power structures) (Meadows, 2008). Addressing city, corporate and individual priorities and beliefs requires higher-level actions directed at policies, which reflect the goals that shape the way the city system operates. Sociological observations show that culture, and consequently urban reliance on fossil fuels, can change if there are enabling social conditions (Poland et al., 2011) that in turn require shifts in worldviews and behaviours.

Health and social theories of behavioural change provide insight not only on the importance of behaviours for health outcomes but also on how to shift behaviour (Davis et al., 2015). Behaviour change theories like Diffusion of Innovation theory emphasise the importance of social networks in communicating and adopting change (Rogers, 2010). However, moving from localised to large-scale social behaviour change requires additional approaches. It is clear that many behaviours are automatic and shaped by socio-ecological factors such as environmental modifications and regulations (Sallis et al., 2012). Current public health thinking supports multi-dimensional system-wide approaches to address complex urban health issues through multi-level policies directed at the political and social factors (Rutter et al., 2017, Craig et al., 2008). This includes bottom-up and top-down actions to change not only structures but also to transfrom deeply entrenched cultures.

A summary of the various theories for change and their application to the urban context and implications for driving transformative change in cities is presented in Table 1. Together these disciplinary perspectives indicate that transformational change for health and sustainability should comprise of integrated multi-scalar systems actors and actions operating across city sectors to change the current political, social-cultural and economic structures swiftly towards equitable management of resources for current and future generations, with a primary focus on improving health within the overarching imperative to achieve urban sustainability.

**Table 1:** Problematising ‘transformational’ and ‘change’ for improving health or environmental sustainability.

Theory	Description	Process	Implications for directing transformative change in cities
Resilience theory	A system’s dynamic capacity to recover, adapt or transform in response to a disturbance. Social ecological resilience focuses on interactions between social, political, economic & environmental components.	People, ecosystems & agencies collectively respond to threats or new opportunities through an adaptive cycle of phases (across space and time scales) to generate reorganisation.	The social-ecological components of a city (physical infrastructure, political and social structures) provide multi-scalar opportunity to deal with uncertainty. The transformative capacity of a city depends on the connection between power, agencies and processes, but it also needs to be flexible. Rigid social, economic or political structures that are disconnected or silo operating resist change.

Social technical studies	Social-technical systems consist of technologies, economies, infrastructure & social structures that enable society to function. Change is a transitional shift to a new social-technical system.	Dynamic processes between agencies act at system levels: (1) niche small-scale spaces for grassroots innovation; (2) regimes or institutional system structures and (3) wider landscape developments	Social functions of cities (governance structures) operate within the social-technical landscape (technological innovations, global forces). Technological innovations for health or sustainability improvements in the city emerge if fostered at the niche level and embedded within policy frameworks.

Complex systems theory	Systems devised of multiple interacting self-organising components. Feedback loops act to stablise or reinforce elements within the system.	Leverage points are places in the system which enable change – though delays or changing quantities, strategies, structures, goals of the system or the greater paradigm of the system	Cities are systems in which social, economic, political and biophysical processes interact and metabolise resources, services, people and ideas. Transformative change requires fundamental levers of the city (city goals, rules, parameters) to change – this requires culture (beliefs) to change.
Social and health behaviour theories	Change in social or health behaviour need to occur at multiple levels (individual risk, social relationships, communities, institutions)	Changing behaviour involves process to shift individual beliefs and attitudes; interpersonal exertions of influence (e.g. self-efficacy), to socio-ecological influences (e.g. environmental modification) and community mobilisation.	Multilevel interventions are needed to target individual, interpersonal, environmental, policy factors to support behaviour change which will motivate culture change such as urban lifestyle changes for redistribution of consumption habits.

## 5 From theory to practice

**Fig. 3:**
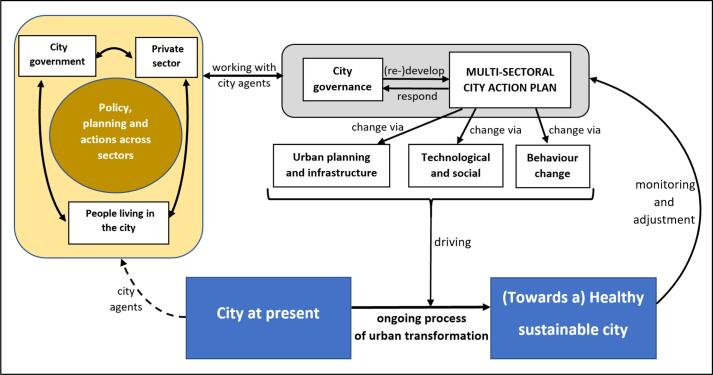
**Framework for achieving urban transformation for health and sustainability.** The figure shows how the agents representing the city government, the private sector and people living in the city (yellow box) collaboratively develop a multi-sectoral action plan via governance processes (grey box), which aims to achieve change via three key mechanisms (urban planning and infrastructure, technological and social change, and behaviour change) as detailed in the main text. The transformation process is ongoing, with monitoring of progress being guiding adjustments to the plan. (For interpretation of the references to colour in this figure legend, the reader is referred to the web version of this article.)

Fig. 3 provides a framework for how transformative change for planetary health may be achieved. First, we contend that the roles of private or public sectors, citizen-led, authoritarian or democratic state agency, will vary in how each can act to mobilise or impede transformation. Second, there will inevitably be variations in environmental health risks or equity issues between cities, depending on their geographical location, region and/or stage of development. There are however a number of levers or mechanisms through which transformational change can advance including city governance to drive urban health outcomes, alongside sustainable urban planning and infrastructure development. Technical and/or social innovation, and behavioural change can also be used to support change.

We reflect on each of these ‘mechanisms’ in the remaining of this section. However, we cannot set out a template for the specific nature of the transformation needed in any particular city because contexts and goals vary. It is clear that to achieve climate change objectives will require fundamental actions to come as close as possible to the elimination of the use of fossil fuels in all sectors. For example, with regard to housing in the UK decarbonising the housing stock, adapted for the current or future climate requires a multi-level intervention (Holmes et al., 2019). In London, all or nearly all of its 3.5 million homes will need to be made very low carbon with upgrades accomplished at the rate of approximately one upgrade every five minutes for the next three decades to meet the 2050 net zero commitment. New homes will need to be designed for a changing climate, ultra-energy efficient and use low- carbon heating. Mitigation and adaptation measures must be integrated in a way that ensures that indoor environmental quality is not compromised. Fuel poor households and those in the socially-rented sector could be prioritised to achieve early gains for wellbeing and health equity but mechanisms need to be found to convert all dwellings to the required standards of energy efficiency. New developments should provide residents with opportunities for sustainable travel, which includes planning neighbourhoods around infrastructure to encourage walking, cycling, the use of public transport and electric vehicles as well as water efficiency performance in homes via measures such as increased metering, compulsory water efficiency labelling, improved behaviours and more ambitious Building Regulation standards. Accomplishing these changes for the whole housing stock of London is a huge undertaking and is the change for just one sector. Plans of equal ambition and comprehensiveness are simultaneously needed in all sectors – in transport, energy, waste, the food system and so on. The scale of investments needed are not widely understood.

### 5.1 Driving transformation through city governance

Many cities operate at a municipality level, however, across a number of often conflicted stakeholder interests which form the governance ‘regime’ in STS discourse. This creates fragmented approaches to integrating urban sustainability and health that often fail to achieve urban transformation (Koch et al., 2017, Koch et al., 2017). Given the interconnection between city sectors, systems-oriented decision making for effective transformation needs to be collaborative, engaging actors across levels and scales to plan and implement actions, rather than working through isolated sectoral structures. This reduces ineffective overlap of authorities and resources that can hinder actions (Hettiarachchi et al., 2018). While city government structures or institutional arrangements are responsible for shaping governing processes, the formulation and implementation of policy also develops through multi-level power and informal networks of actors inside and outside government (Grandin et al., 2018). This integrative, flexible city management is necessary to manage resources efficiently (Childers et al., 2014). Collaborative decision-making promotes the alignment of objectives to co-create sustainable healthy policies and plans, with input from all actors involved in the city processes underpinning decision making (Linnenluecke et al., 2017).

Effective policy implementation is essential for population level change. There has been movement away from command and control policies in many societies, towards the use of innovative approaches in public policy, flexible regulation and instruments, such as incentive programs, tradable permits, pollution charges, eco-audits, and voluntary agreements. These are sometimes considered more effective, non-coercive and efficient instruments of environmental policy than top-down authoritarian approaches (Kochtcheeva, 2008). However, changing deep-rooted cultural norms to prevent lifestyle-related chronic diseases or promoting sustainable practices across competitive markets generally requires strong and integrated intervention and governance across various interest groups.

Copenhagen is an exemplar for how the municipality has worked with various stakeholders to integrate plans for climate change mitigation and the promotion of health in everyday life. The city has long established policy for the decarbonisation of the transport and energy sectors, including provision of bicycle and public transportation infrastructure and urban energy use focusing on wind power and other renewable energy. Moreover, the municipality looks at taking into account emission produced outside the city which contribute to the consumption of goods within the city (i.e. consumption-related emissions). This includes policy targets and planning initiatives across multiple sectors (business and household energy use, energy production, transport and city administration) which does provide potential co-benefits for health such as physical activity and pollution reduction, as well as delivering wider wellbeing and equity benefits to Copenhagen’s hinterlands. Copenhagen’s municipal government is powerful and has been able to make strategic decisions that span across decades and mayoral mandates. This is supported by consistent and long-standing collaborations across levels of government, political parties and sectors of society. Such strategic, multi-level and multi-sectoral governance will be necessary to achieve systemic change for sustainability and health in cities.

### 5.2 Technological and social innovations

System innovations are about using technology alongside other city components (infrastructure, supply networks, regulatory structures) to shift current practices (Geels, 2005). Private business plays an important role in enabling such changes to occur through technological developments such as renewable energy technologies (e.g. photovoltaic panels), green innovation technologies (e.g. waste water treatment technologies) or smart city technologies (e.g. street light sensors, smart garbage bins sending data to waste management systems). Opportunities to use smart technologies for social, environmental and health initiatives are promising, although they require nurturing (De Jong et al., 2015, Evans et al., 2019). Smart city technologies have the potential for improving living standards, administrative and economic efficiency, and improving urban processes such as in electricity use and waste processing (Evans et al., 2019), yet they have the risk of widening inequities if the planning and policy infrastructure and coordination is weak as observed in India’s Smart Cities Movement (Praharaj et al., 2018). In Japan, smart city initiatives have applied smart technologies to energy supply and tackling health and social issues including population aging and health monitoring, and more recently, wellbeing (information provision and behaviour change incentives) (Trencher and Karvonen, 2019). Digitalisation and big data have the potential to change how people in cities interact with each other, the surrounding environment and urban infrastructure. However, while the Internet, geospatial mapping, citizen science, sensor and other urban data sources are being used, their application to urban health and social issues needs to expand. Also, if technological innovation improves, resource use may result in increased consumption if sustainable goals for both climate mitigation and adaptation are not emphasised. Such innovations must therefore be implemented in the context of carbon abatement and other resource constraining strategies.

### 5.3 Urban planning and infrastructure development

An important mechanism at the hands of cities and local governments is their planning powers. Most theories and conceptual frameworks in urban studies recognise the role that spatial planning plays in orchestrating sustainability and health policy and action at the city level and, more importantly, across spatial scales. While planning approaches vary across regions, most cities have some degree of control over spatial or land-use decisions, which they can use to create more sustainable and healthier built environments via encouraging people to be physically active, creating opportunities for social interaction, providing access to green spaces, and minimising exposure to air pollution and other pollutants (PHE, 2017). Examples of cities using their planning powers to promote health include the Active City programme in Amsterdam to encourage designers and urban planners to create active spaces for all, where neighbourhoods with the highest levels of need and deprivation are targeted. However, transformative planning needs not only address neighbourhood issues but also macro-scale challenges. Transformative macro-scale actions include decentralisation of business districts. Neighbourhood scale planning (i.e. green space, neighbourhood walkability) are arguably actions that can be most easily implemented under the current structures, but do not generally have the capacity for change at scale and often fail to address equity and social justice issues (Hughes, 2015). This is why planning for transformational change needs to involve a range of stakeholders, including representatives from communities and researchers (Linnenluecke et al., 2017). In Africa, rapid urbanisation threatens to have a negative effect on sustainability and health outcomes, particularly due to unplanned development; with poor quality and limited employment opportunities, poverty, water and sanitation (including waste management) of major concern (Cobbinah et al., 2015).

### 5.4 Social behaviour

Changing unsustainable or unhealthy lifestyles requires actions to address social norms, which in turn and as argued by social theories of behavioural change can impact on health outcomes. Collective and individual actions are likewise essential for achieving sustainable behaviour transformation across society (Messerli et al., 2019). Social inertia can occur at the individual, institutional or societal level: from information deficits; failure to integrate knowledge into individual behaviour and organisational practice and shift in power structures. This means that behaviour change interventions need to focus more on social and political factors determining individual and organisational behaviour, particularly in areas such as diet, energy use, travel and waste. Social movements on climate change like youth-led activism groups and civil resistance groups are more organised, globally and unlike previous movements, focused on global and intergenerational justice. As a result, community concern for climate change is reportedly growing. These movements could create a social tipping point, but still need to overcome resistance and counter movements aiming to maintain the status quo (Praharaj et al., 2018), and have not yet reached a level of public urgency to push decisive political action anywhere to-date. Nudge theory has gained widespread interest as a way to shift behaviour in public policy, including public health and environment policy as an alternative to regulation policies (Hansen et al., 2016). Policy tools include simplification and framing of information; changes to the physical environment; changes to default policies; and the use of social norms (Lehner et al., 2016). The success of these strategies is largely context-specific (different barriers require different interventions) and must complement rather than substitute policy instruments (i.e. laws and regulations) (Lehner et al., 2016). Whatever the effect of these strategies, there is no substitute for enlightened leadership which is probably a crucial ingredient in every context. Such leadership is more likely to emerge with better understanding of the nature of the evidence relating to the challenges for planetary and population health and of possible solutions – which means an important role for science in informing policy-makers and the public alike.

### 5.5 Scientific evidence as a driver for innovative action

Currently there is a lack of integration between each of these potential mechanisms for achieving and/ or implementing transformative change in cities. Scientific evidence plays an important role in supporting complex urban decisions and joining efforts of city operations, community and industry action, particularly if it is co-produced with ‘end-users’ and embedded into the culture of decision-making. However, the potential for effective knowledge translation, such as by bringing researchers together with policymakers to explore urban challenges and potential solutions, has been largely overlooked (Oliver et al., 2014, Sallis et al., 2016). Science can help forecast and model the effects of potential policies, interventions and assess where innovation is of particular value, building scenarios to assess the long-term effects and potential negative consequences from a systems perspective (McCormick et al., 2013, Meadows, 2008, Linnenluecke et al., 2017). Co-created experimental approaches such as Urban Living labs gather input from research, public and private sectors to design and test social, political, and environmental or health interventions in the urban context. This is an innovative type of knowledge co-creation, which can contribute to reconfiguring conditions, resources and people (Hansen et al., 2016, Sallis et al., 2016). Central to the process of transformational change in cities is the need to partner city decision makers and scientific research drawing together expertise and knowledge to (1) build consensus about the problem and possible solutions (2) agree on general objectives and (3) develop ongoing collaboration and mutual understanding. This approach will also reduce the disconnect between academia and real world actions on global challenges (Black et al., 2018).

## 6 Conclusion

There is an unrealised potential to integrate sustainability and health at the city level to achieve wider planetary health in the face of multiple environmental, economic and social changes that threaten to undermine human progress. Change needs to occur at a pace and scale not previously undertaken. This requires new ambitious and integrative approaches that uses translational science and integrates the urban structures and processes to drive change in cities to improve health, enhance resilience to environmental change and reduce the environmental footprint of cities.

## Declaration of Competing Interest

The authors declare that they have no known competing financial interests or personal relationships that could have appeared to influence the work reported in this paper.
