# Supplementary material for: Transforming cities for sustainability: A health perspective
Source: Environ Int. 2021 Feb;147:106366. doi: 10.1016/j.envint.2020.106366 (PMC8543068; doi:10.1016/j.envint.2020.106366)
Supplement: Supplementary Table S.1 [file mmc1.docx]

**Table S.1. Urban challenges and opportunities for integrated policy intervention in cities**

| **Sectors and urban form** | **Urban environmental challenges** | **Urban health challenges** | **Goals contributing to transformation via actions on the  environment & health**[1-5] |
| --- | --- | --- | --- |
| Buildings | GHG emissions  Climate change-related impacts (droughts, floods, heatwaves)  Air quality: radon & VOC emissions  Sustainable resources  Housing availability and affordability | Infectious diseases  Heat stress  Acute and chronic diseases associated with pollution exposure (noise, air, soil).  Mental health  Injury & violence  Equitable access to shelter | Carbon emission reductions  Climate change adaptation and resilience  Sustainable infrastructure  Equity & justice  Minimised exposure to pollution & hazards  Reduction in and redistribution of consumption |
| Water and sanitation | Water quantity and quality | Infectious diseases  Equitable access to water | Reduction in and redistribution of consumption  Equity & justice  Minimised exposure to pollution & hazards |
| Transport | GHG emissions  Traffic congestion  Traffic noise  Urban sprawl  Urban Heat island | Acute and chronic diseases associated with lack of physical activity and pollution exposure (air, noise)  Mental health  Traffic injuries  Equitable mobility | Sustainable infrastructure  Reduction in and redistribution of consumption  Carbon emission reductions  Equity & justice  Minimised exposure to pollution & hazards |
| Natural environments including green space | Loss of ecological habitat & biodiversity  Adequate green spaces | Mental health  Acute and chronic diseases associated with lack of physical activity  Gut microbiome  Asthma & allergy  Equitable access to green spaces | Sustainable infrastructure  Reduction in and redistribution of consumption  Equity & justice |
| Energy supply and use | GHG emissions  Energy supply, use & efficiency | Acute & chronic diseases associated with air quality.  Heat stress  Equitable access to energy | Carbon emission reduction  Sustainable infrastructure  Reduction in and redistribution of consumption  Social equity & justice |
| Food supply and consumption | GHG emissions  Local food security  Food & packaging waste  Unsustainable agriculture & production | Health outcomes related to malnutrition in all its forms.  Equitable access to healthy food | Sustainable infrastructure  Reduction in and redistribution of consumption  Equity & justice  Carbon emission reduction |

 Note: Key environmental and health challenges and overarching goals included are illustrative and not an exhaustive list. The table was derived from an overview of urban health and environmental challenges and primary goals collated from existing literature and a workshop 6-7 September 2018 with experts from across multiple disciplines, policy and industry representatives for the Wellcome Trust funded CUSSH project (Complex Systems for Sustainability and Health). Experts included research disciplines of Public Health, Environmental Science, Architecture, Building and urban planning, economics, Law, Ecology, Transport, Behavioural science, Engineering, and Systems dynamics. City representatives from the city of Kisumu, Nairobi, Rennes Metropole and European WHO Healthy cities, China Centre for Disease Control, BuroHappold, C40 cities, Ecological sequestration Trust and Public Health England.

References

1. Gao, J., et al., *Public health co-benefits of greenhouse gas emissions reduction: A systematic review.* Science of the Total Environment, 2018. **627**: p. 388-402.

2. Whitmee, S., et al., *Safeguarding human health in the Anthropocene epoch: report of The Rockefeller Foundation–Lancet Commission on planetary health.* The Lancet, 2015. **386**(10007): p. 1973-2028.

3. Edenhofer, O., *Climate change 2014: mitigation of climate change*. Vol. 3. 2015: Cambridge University Press.

4. Rydin, Y., et al., *Shaping cities for health: complexity and the planning of urban environments in the 21st century.* The Lancet, 2012. **379**(9831): p. 2079-2108.

5. World Health Organization, *Health as the pulse of the new urban agenda: Unite Nations conference on housing and sustainable urban development, Quinto, October* 2016, WHO: Geneva.
